# Targeting Aquaporin-5 by Phosphodiesterase 4 Inhibition Offers New Therapeutic Opportunities for Ovarian Ischemia Reperfusion Injury in Rats

**DOI:** 10.1007/s43032-024-01496-w

**Published:** 2024-03-07

**Authors:** Ayse Bozkurt, Zeynep Karakoy, Pelin Aydin, Bengul Ozdemir, Erdem Toktay, Zekai Halici, Elif Cadirci

**Affiliations:** 1 Faculty of Pharmacy, Department of Pharmacology, Van Yuzuncu Yil University, Van, Turkey; 2 Faculty of Pharmacy, Department of Pharmacology, Erzincan Binali Yildirim University, Erzincan, Turkey; 3 Faculty of Medicine, Department of Pharmacology, Ataturk University, Erzurum, 25240 Turkey; 4 Department of Anesthesiology and Reanimation, Educational and Research Hospital, Erzurum, Turkey; 5 Faculty of Medicine, Department of Histology and Embryology, Kafkas University, Kars, Turkey; 6 Clinical Research, Development and Design Application and Research Center, Ataturk University, 25240 Erzurum, Turkey

**Keywords:** Ischemia, Ovary, PDE4, Reperfusion, Rolipram

## Abstract

This study aimed to examine the effect of Phosphodiesterase 4 (PDE4) inhibition on Aquaporin-5 (AQP5) and its potential cell signaling pathway in the ovarian ischemia reperfusion (OIR) model. Thirty adult female rats were divided into five groups: Group 1; Control: Sham operation, Group 2; OIR that 3 hour ischemia followed by 3 hour reperfusion, Group 3; OIR + Rolipram 1 mg/kg, Group 4; OIR + Rolipram 3 mg/kg, Group 5; OIR + Rolipram 5 mg/kg. Rolipram was administered intraperitoneally to the rats in groups 3-4 and 5 at determined doses 30 minutes before reperfusion. From ovary tissue; Tumor necrosis factor-a (TNF-α), Cyclic adenosine monophosphate (cAMP), Nuclear factor kappa (NF-κB), Interleukin-6 (IL-6), Phosphodiesterase 4D (PDE4D), Mitogen-activated protein kinase (MAPK) and AQP5 levels were measured by ELISA. We also measured the level of AQP5 in ovary tissue by real-time reverse-transcription polymerase chain reaction (RT-PCR). In the OIR groups; TNF-α, NF-κB, IL-6, MAPK inflammatory levels increased, and cAMP and AQP5 levels decreased, which improved with the administration of rolipram doses. Also histopathological results showed damaged ovarian tissue after OIR, while rolipram administration decrased tissue damage in a dose dependent manner. We propose that the protective effect of PDE4 inhibition in OIR may be regulated by AQP5 and its potential cell signaling pathway and may be a new target in OIR therapy. However, clinical studies are needed to appraise these data in humans.

## Introduction

Ovarian torsion is a gynecological emergency that can affect women of all ages, particularly women of reproductive age, and leads to ovarian ischemia reperfusion (OIR) injury through detorsion [1]. However, reperfusion that occurs as a result of detorsion of the ovaries has some systemic and local effects. Thus, with the ischemic damage caused by torsion, reperfusion damage consist of in the ovaries and this causes an ischemia/reperfusion (I/R) issue [2]. This is accompanied by the release of large amounts of free radicals, with oxidative tissue damage having a significant impact on the damage caused by OIR [3] In cases of ovarian torsion, an I/R injury may develop due to the release of free radicals and reactive oxygen species (ROS) during the detorsion process [4]. Excessive production of ROS, which are hydroxyl radicals, hydrogen peroxide and superoxide radicals, is one of the complications of I/R that causes serious damage to reproductive tissues [5]. Ovarian torsion occurs with a frequency of 2.7-7.4%, depending on the series, and most frequently during the reproductive years, and patients are usually in their mid-20s [6] and occur with a frequency of about 15% among infants and children [7].Therefore, early diagnosis and treatment is important in this disease and increases the chance of preserving ovarian function and fertility [8]. Phosphodiesterases (PDEs) are enzymes consisting of 11 subtypes PDE1-PDE11 and hydrolyzing two secondary messengers, cyclic guanosine mono-phosphate (cGMP) and cyclic adenosine monophosphate (cAMP) [9]. PDEs are involved in the regulation of many physiological mechanisms including gene expression, cell proliferation, differentiation, apoptosis, metabolism, visual transduction, inflammation [10]. PDE4 is one of the PDE families responsible for the hydrolysis of cAMP. This is accompanied by the release of large quantity of free radicals, with oxidative tissue damage having a significant impact on the damage caused by OIR and separated into four subtypes PDE4A/4B/4C/4D [11]. PDE4 is commonly found in immune and inflammatory cells such as neutrophils, macrophages and monocytes [12]. Selective inhibition of PDE4 contributes to the suppression of many aspects of the inflammatory response by increasing the cAMP content in many immunomodulatory and inflammatory cells [13]. Few studies have reported signals of activation of inflammatory cells due to an increase in cAMP [14]. However, the importance of activation of cAMP signaling on the overall response of inflammatory cells is mostly unknown [15]. Rolipram is known as a selective PDE4 inhibitor has been shown in previous experimental studies to suppress local increases in vascular permeability and neutrophil recruitment following I/R injury in a dose-dependent manner (1 to 10 mg/kg) and increases intracellular cAMP levels in many tissues and cell types [16].

Aquaporins (AQPs) not only regulate transepithelial fluid transport across membranes, but are also involved in the regulation of key events crucial for the inflammatory response. Several studies have shown that AQPs including AQP5, have important roles in inflammatory diseases. This clearly indicates that AQPs may be potential targets in inflammatory diseases including I/R [17, 18]. As there is an urgent need for further preventive treatment to preserve ovary tissue during OIR injuries, we considered examining the different pathways that cause such damage. Hence, this study aimed to investigate the relationship between PDE4, which is widely present in inflammatory cells, and AQP5 in the development of inflammation in OIR injury. Therefore, we tried to appraise for the first time the role of PDE4 inhibitor rolipram in an OIR-induced injury model and its relationship in distinct pathways mediating this injury to IL-6/ NF-κB/TNF-α /MAPK and cAMP/AQP5.

## Materials and Methods

### Chemicals

Rolipram (R0110) was purchased from TCI (Zwijndrecht, Belgium). TNF-α (Catalog #201-11-0765), IL-6 (Catalog #201-11-0136), cAMP (Catalog #201-11-029), AQP5 (Catalog #201-11-0570), MAPK (Catalog#201-11-1064), NF-κB (Catalog#201-11-0288), cAMP Spesific (PDE4D) (Catalog #201-12-4539) ELISA kits were obtained from SunRed (Shanghai, China) and AQP5 250 rxn (Rn00562837_m1) was purchased from Thermo Fisher Scientific (Budapest, Hungary).

### Animals

Wistar albino rats, approximately 12 weeks old and weighing 220-240 grams, were kept in steel cages under standard conditions (7am-8pm light period, 55% relative humidity, and 21±2 °C) throughout the experiments, and were given tap water and standard pellet feed ad libitum. All animal protocols and care were confirmed by Experimental Animal Ethics Committee of Ataturk University (13.01.2023/13).

### Preparation and Treatment

Rats were separated into five groups (*n*=6), fasted for 24 hours, and administered the following chemicals:

- Group 1: Control: Sham operation performed.
- Group 2: OIR
- Group 3: OIR + Rolipram (1 mg/kg) single *i.p.* dose 30 min before reperfusion
- Group 4: OIR + Rolipram (3mg/kg) single *i.p.* dose 30 min before reperfusion
- Group 5: OIR + Rolipram (5mg/kg) single *i.p.* dose 30 min before reperfusion

#### Surgical Protocol

All procedures were performed under sterile conditions and general anesthesia. For general anesthesia, each rat was injected intraperitoneally with 5 mg/kg xylazine hydrochloride and 45 mg/kg ketamine hydrochloride. The abdominal skin was shaved and cleaned with 10% povidone iodine. The lower abdomen was opened with a 2 cm midline incision and bilateral ovaries were exposed. After the ovaries were identified, ischemia was created with atraumatic vascular clamps (Bulldog clamps) just below the left ovary and the incisions were closed with 4-0 silk sutures. After a 3 hours period of ischaemia, the atraumatic vascular clamps were removed and reperfusion was achieved for 3 hours. In the control group, only laparotomy was performed. 1, 3 and 5 mg/kg rolipram [16, 19, 20] was administered intraperitoneally to the rats in groups 3, 4 and 5 respectively, 30 minutes before reperfusion. To protect the rats from hypothermia, the operating table was heated using a lamp from above and a heater from below. During the waiting period, the incision line in the abdominal area was closed with 3-0 silk suture. In the final stage of the experiment, all rats were sacrificed, their ovaries were removed and stored in %10 formaldehyde for histopathological and molecular examinations [21].

### Molecular Measurements

#### Measurement of Ovary Tissue

After the surgical procedures, about 100 mg of ovary tissue was homogenized in 2 ml of phosphate-buffered saline (PBS) in eppendorf tubes using a homogenizer (TissueLyser II by QIAGEN), and then centrifuged. AQP5, NF-κB, MAPK, PDE4D, cAMP, IL-6, and TNF-α levels from the obtained supernatants were measured by ELISA method in Epoch Spectrophotometer System and Take3 Plate device. An equation was obtained from the absorbance of the standards by plotting a standard curve. Linear AQP5, NF-κB, MAPK PDE4, cAMP, IL-6, and TNF-α concentrations were calculated according to this equation as previously described [22]. All ELISA kits were used according to manufacturer’s directives and measured using a BioTek Epoch Microplate Spectrophotometer.

### Evaluation Of AQP5 With real-time reverse-transcription polymerase chain reaction (RT-PCR)

#### Ribonucleic Acid(RNA) Extraction from Ovary Tissue

Rat ovary tissue was individually weighed, homogenized in the Tissue Lyser II device (350 µl of RLT buffer was added to 20-30 mg of tissue) and RNA was extracted in the QIAcube RNA isolation tool. Total RNA was isolated in the Qiaqube RNA isolation tool using the RNeasy Mini Kit. Total mRNA quantity was measured by nano drop spectrophotometry at 260/280 nm. The resulting RNA was stored -80 °C under the necessary conditions.

### Reverse Transcriptase Reaction and Synthesis of Complementary DNA (cDNA)

Using the High Capacity cDNA Reverse Transcription Kit enzyme, cDNA synthesis was performed from total RNA. Each reaction was actualized with 10 μl of RNA and cDNA synthesis was achieved with Veriti 96 Well Thermal Cycler according to the specified temperature values. The quantity of cDNA was determined by nano drop spectrophotometry and stored at -20˚C. For cDNA synthesis, 10 μl total RNA; 10 X RT 30 Buffer 2 μl; MultiScribe 10 X RT Random Primers 2 μl; Reverse Transcriptase 1 μl; 25 X dNTPs mix 0.8 μl; DEPC-H2O 4.2 μl) were used [23].

### Quantitative Detection of AQP5 mRNA Expressions by RT-PCR

The relative expression analyses of AQP5 were performed with the StepOnePlus Real Time PCR System (Applied Biosystems) using cDNA synthesized from rat ovary RNA. The real-time quantitative reverse transcriptase PCR was run using Primer Perfect Probe mix, TaqMan Probe-based technology (Primer Design Ltd., Southampton, UK), and the results were expressed as the relative-fold change in expression as compared with that in the control animals. Specific primer were used for rat gene transcripts Rn00562837_m1. The gene expression levels were normalized using β-actin (Rn00667869_m1) as a housekeeping gene. For each tissue, triplicate determinations were performed in a 96-well optical plate for all parameters using 9 μL of cDNA (100 ng), 1 μL of Primer Perfect Probe mix, and 10 μL of QuantiTect Probe PCR Master mix (Qiagen) in each 20 ml reaction. The plates were heated for 2 min at 50 °C and 10 min at 95 °C, followed by 40 cycles of 15 s at 94 °C and 60 s at 60 °C. All data are expressed as the fold-change in expression as compared with the expression in other animal groups, using the 2−ΔΔCt method [24, 25].

### Histopathological Analyzes

Ovary tissue samples were fixed in 10% formalin solution for 72 hours. Subsequently, routine tissue processing was performed. Tissues were first washed in running water for 20 minutes and formalin was removed. Then, water was removed from the tissues by passing one hour through increasing alcohol series (50, 60, 70, 80, 96 and 99). Then, the tissue samples were made transparent by passing through the xylene series 3 times for 15 minutes. Finally, the tissues were thrown into molten paraffin, which was kept in an oven at 60°C, and 2 changes were kept for 1 hour each, and the paraffin was absorbed into the tissue. Tissue samples were then blocked for sectioning. Paraffin blocks were cut with a microtome at a thickness of 5 microns. Sections were stained with hematoxylin and eosin stains. The sections were examined and photographed with an Olimpus CX 21 camera-attached microscope. Finally, the images were brought together with Photoshop CS5.

### Statistical Analyses

All the results were expressed as mean standard deviation (SD) of the mean. The numerical data regarding molecular and biochemical analyses were subjected to one-way analysis of variance (ANOVA) using the IBM SPSS Statistics 20.0 software program (SPSS Inc., Chicago, IL). Differences among the groups were determined using the Tukey test and were considered to be significant when the p values was less than 0.05 in a 95% confidence interval.

## Results

### Molecular Findings

#### Inflammatory Cytokine ELISA Results

**Fig. 1:**
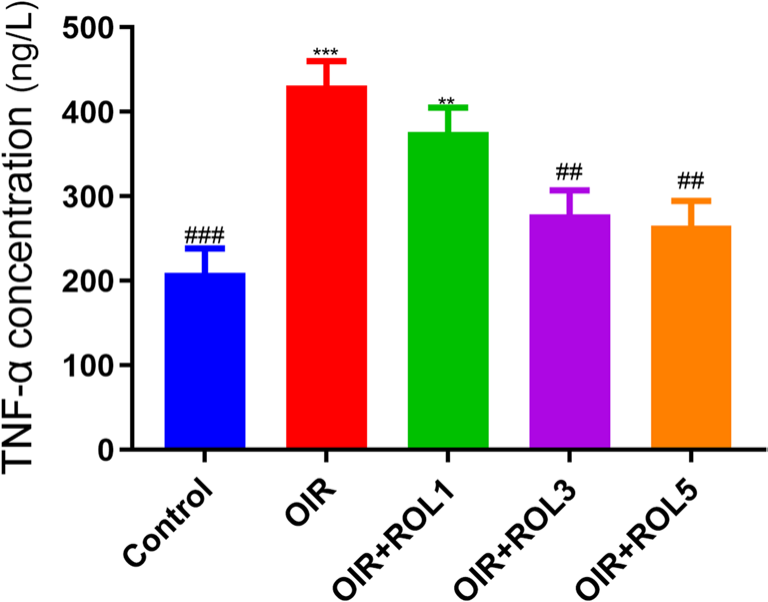
Effects of Rolipram (ROL) on TNF-α levels in ovarian ischemia-reperfusion injury. *p<0.05 **p<0.01 ***p<0.001 indicate comparison with control group. #*p*<0.05, ##*p*<0.01 and ###*p*<0.001 indicate the comparison by OIR group according to Tukey test. Results are given as mean ± standard deviation. ROL 1: Rolipram 1 mg/kg, ROL 3: Rolipram 3 mg/kg, ROL 5: Rolipram 5 mg/kg

TNF-α levels were measured in ovary tissue. TNF-α levels were significantly increased in the ovary tissue of OIR induced rats compared to the control group (*p*<0.001). With the application of PDE4 inhibitor rolipram, TNF-α levels were significantly decreased in the OIR+ROL3, OIR+ROL5 group compared to the OIR group (*p*<0.01) (Fig. 1).

**Fig. 2:**
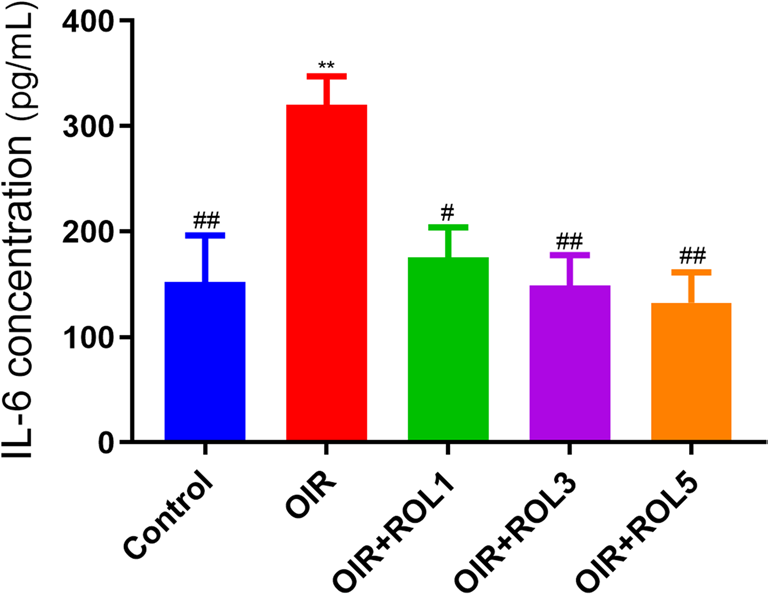
Effects of Rolipram (ROL) on IL-6 levels in ovarian ischemia-reperfusion injury. *p<0.05 **p<0.01 ***p<0.001 indicate comparison with control group. #*p*<0.05, ##*p*<0.01 and ###*p*<0.001 indicate the comparison by OIR group according to Tukey test. Results are given as mean ± standard deviation. ROL 1: Rolipram 1 mg/kg, ROL 3: Rolipram 3 mg/kg, ROL 5: Rolipram 5 mg/kg

IL-6 levels were measured in ovary tissue. IL-6 levels in the ovary tissue of OIR-induced rats were significantly increased compared to the control group (*p*<0.01). With the administration of PDE4 inhibitor rolipram, IL-6 levels decreased in a dose-dependent manner compared to the OIR group. Namely, while it decreased significantly in the OIR+ROL1 group (*p*<0.05); It was evaluated that OIR+ROL3 (*p*<0.01), OIR+ROL5 (*p*<0.01) decreased significantly (Fig. 2).

### AQP5 ELISA Result

**Fig. 3:**
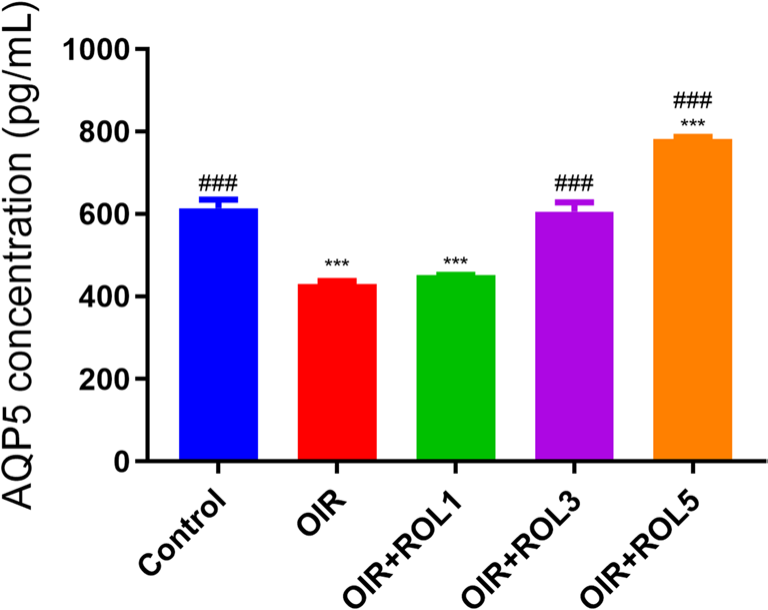
Effects of Rolipram (ROL) on AQP5 levels in ovarian ischemia-reperfusion injury. *p<0.05 **p<0.01 ***p<0.001 indicate comparison with control group. #*p*<0.05, ##*p*<0.01 and ###*p*<0.001 indicate the comparison by OIR group according to Tukey test. Results are given as mean ± standard deviation. ROL 1: Rolipram 1 mg/kg, ROL 3: Rolipram 3 mg/kg, ROL 5: Rolipram 5 mg/kg

In our study, AQP5 levels were measured in ovary tissue. AQP5 levels were significantly decreased in the ovary tissue of OIR induced animals compared to the control group (*p*<0.001). No significant increase was observed in the OIR+ROL1 group compared to the OIR group. In the OIR+ROL3 group, AQP5 levels were increase compared to the OIR group (*p*<0.001). In the OIR+ROL5 group in which the highest dose of rolipram was applied, AQP5 levels showed the highest increase compared to the OIR group (p<0.001) (Fig. 3).

### NF-κB ELISA Result

**Fig. 4:**
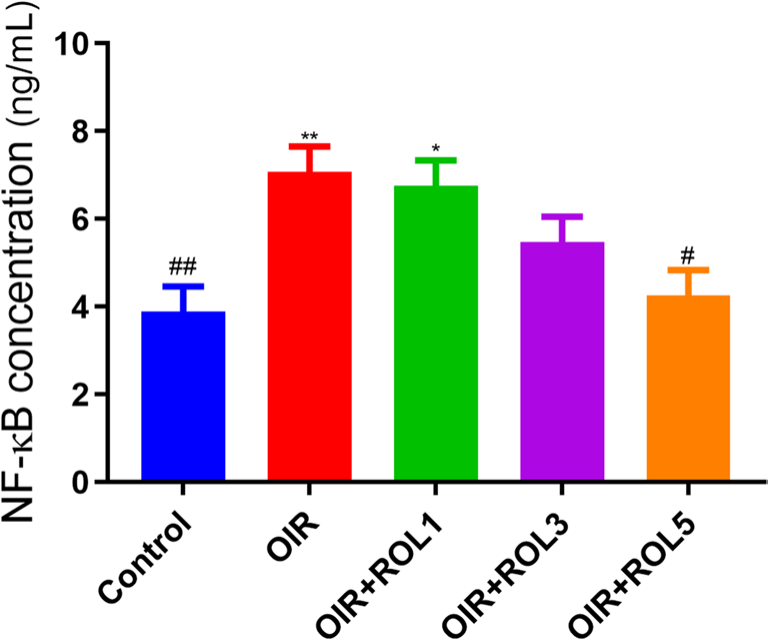
Effects of Rolipram (ROL) on NF-κB levels in ovarian ischemia-reperfusion injury. *p<0.05 **p<0.01 ***p<0.001 indicate comparison with control group. #*p*<0.05, ##*p*<0.01 and ###*p*<0.001 indicate the comparison by OIR group according to Tukey test. Results are given as mean ± standard deviation. ROL 1: Rolipram 1 mg/kg, ROL 3: Rolipram 3 mg/kg, ROL 5: Rolipram 5 mg/kg

NF-κB signal was measured in ovary tissue in our study. NF-κB levels were significantly increased in the ovary tissue of OIR induced animals compared to the control group (*p*<0.01). With the application of PDE4 inhibitor rolipram, a significant decrease was observed in NF-κB levels in the OIR+ROL5 group compared to the OIR group (*p*<0.05) (Fig. 4).

### MAPK ELISA Result

**Fig. 5:**
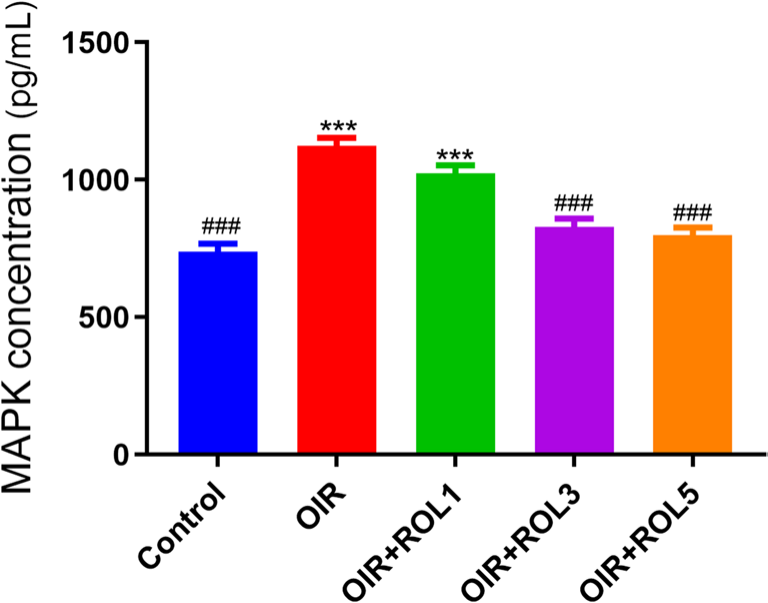
Effects of Rolipram (ROL) on MAPK levels in ovarian ischemia-reperfusion injury. *p<0.05 **p<0.01 ***p<0.001 indicate comparison with control group. #*p*<0.05, ##*p*<0.01 and ###*p*<0.001 indicate the comparison by OIR group according to Tukey test. Results are given as mean ± standard deviation. ROL 1: Rolipram 1 mg/kg, ROL 3: Rolipram 3 mg/kg, ROL 5: Rolipram 5 mg/kg

MAPK levels were measured in ovary tissue. A significant increase was observed in MAPK levels in the ovary tissue of OIR-induced animals compared to the control group (*p*<0.001). With the application of PDE4 inhibitor rolipram, a dose-dependent decrease in MAPK levels was observed in the OIR+ROL3, OIR+ROL5 groups compared to the OIR group (*p*<0.001) (Fig. 5).

### PDE4D ELISA Result

**Fig. 6:**
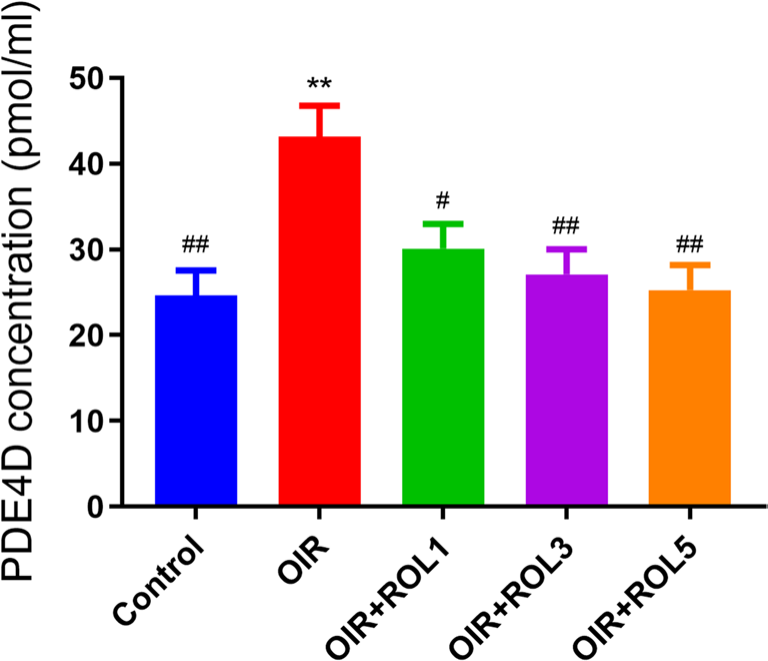
Effects of Rolipram (ROL) on PDE4D levels in ovarian ischemia-reperfusion injury. *p<0.05 **p<0.01 ***p<0.001 indicate comparison with control group. #*p*<0.05, ##*p*<0.01 and ###*p*<0.001 indicate the comparison by OIR group according to Tukey test. Results are given as mean ± standard deviation. ROL 1: Rolipram 1 mg/kg, ROL 3: Rolipram 3 mg/kg, ROL 5: Rolipram 5 mg/kg

PDE4D levels were measured in ovary tissue. PDE4D levels in the ovary tissue of OIR induced animals were significantly increased compared to the control group (*p*<0.01). Administration of PDE4 inhibitor rolipram to OIR treated animals caused a dose-dependent decrease in PDE4D level. Namely; A significant decrease was observed in the OIR+ROL3 (*p*<0.01) and OIR+ROL5 (*p*<0.01) groups, while a decrease was observed in the OIR+ROL1 (*p*<0.05) group compared to the OIR group (Fig. 6).

### cAMP ELISA Result

**Fig. 7:**
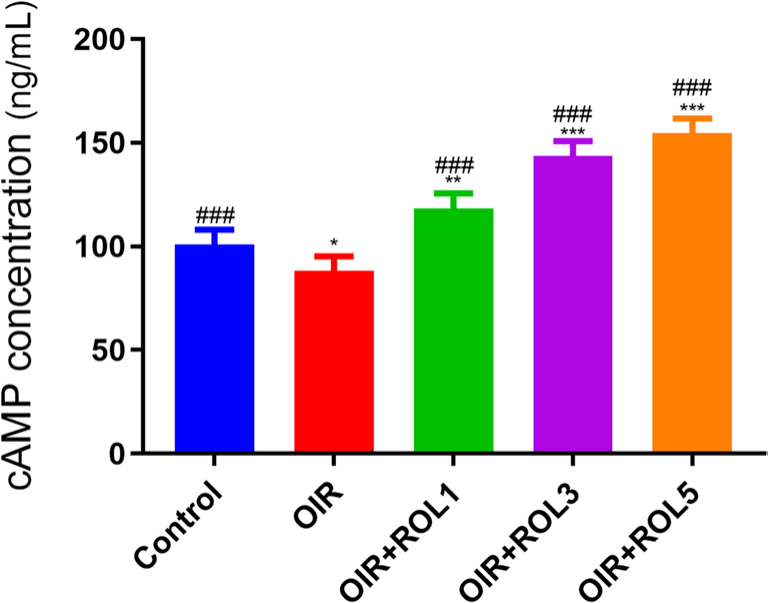
Effects of Rolipram (ROL) on cAMP levels in ovarian ischemia-reperfusion injury. *p<0.05 **p<0.01 ***p<0.001 indicate comparison with control group. #*p*<0.05, ##*p*<0.01 and ###*p*<0.001 indicate the comparison by OIR group according to Tukey test. Results are given as mean ± standard deviation. ROL 1: Rolipram 1 mg/kg, ROL 3: Rolipram 3 mg/kg, ROL 5: Rolipram 5 mg/kg

cAMP levels were measured in ovary tissue. In the OIR group, cAMP levels were significantly decreased compared to the control group (*p*<0.05). With the administration of the PDE4 inhibitor rolipram, cAMP levels increased significantly in the OIR+ROL1 (*p*<0.01), OIR+ROL3 (*p*<0.001), OIR+ROL5 (*p*<0.001) groups compared to the OIR group (Fig. 7).

### Expression of AQP5 in Over Tissues

**Fig. 8:**
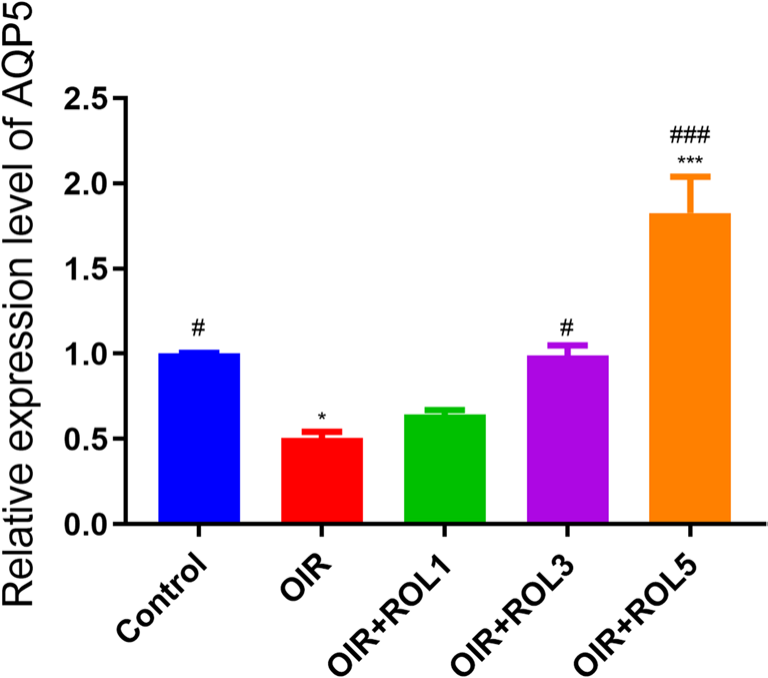
Effects of Rolipram (ROL) on AQP5 mRNA expression levels in ovarian ischemia-reperfusion injury. *p<0.05 **p<0.01 ***p<0.001 indicate comparison with control group. #*p*<0.05, ##*p*<0.01 and ###*p*<0.001 indicate the comparison by OIR group according to Tukey test. Results are given as mean ± standard deviation. ROL 1: Rolipram 1 mg/kg, ROL 3: Rolipram 3 mg/kg, ROL 5: Rolipram 5 mg/kg

When AQP5 mRNA Expression levels in the ovary tissue were examined in the OIR model, it was observed that there was a significant decrease in the OIR group compared to the control group (*p*<0.05). With the application of PDE4 inhibitor rolipram, AQP5 mRNA expression levels were not observed to increase significantly in the OIR+ROL1 group compared to the OIR group, whereas in the OIR+ROL3 (*p*<0.05) and OIR+ROL5 (*p*<0.001) groups, AQP5 mRNA expression levels were observed to increase compared to the OIR group (Fig. 8).

### Histopathological Results

**Fig. 9:**
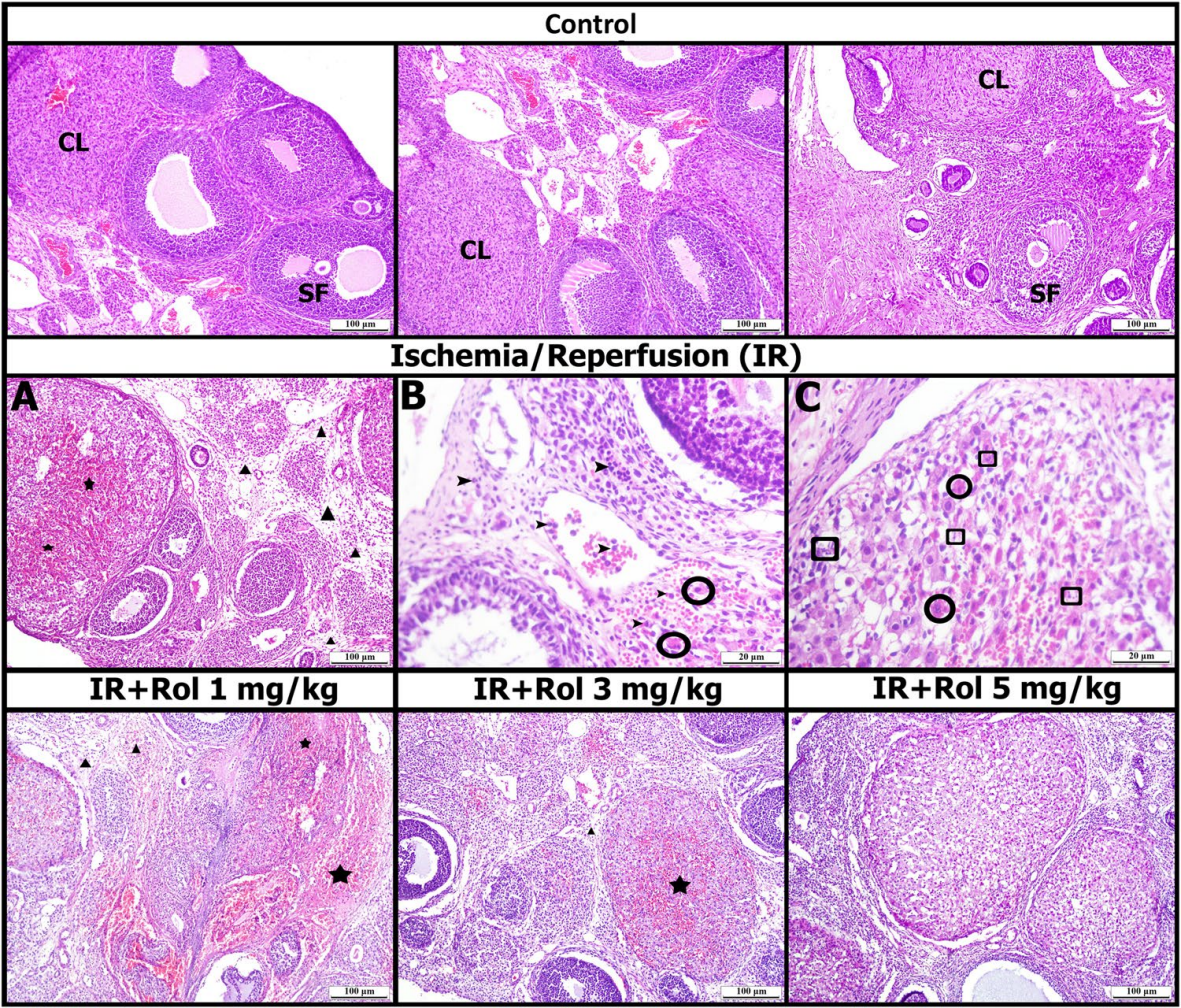
Histopathological findings (Ischemia and Reperfusion: IR, Rol: Rolipram, Star: Hemorrhage, Triangle: Edema, Round Ring: necrotic cell, Square: Apoptotic cell, Arrowhead: Inflammatory cell, CL: Corpus luteum, SF: Secondary follicle)

The medulla and cortex of the ovary were examined in the control group. Control-looking primary, secondary follicles and copus luteum were observed in the cortex. Normal-looking blood vessels were observed in the medulla (Fig. 9, Table 1).

**Table 1:** Histopathological findings scoring

GROUPS	Hemorrhage	Edema	Inflammatory Cell	Necrotic Cell
Control	-	-	-	-
OIR	++	++	++	++
OIR+ROL 1	++	++	++	+
OIR+ROL 3	+	+	+	-
OIR+ROL 5	-/+	-	-	-

Semi-quantitative scoring was used to evaluate the histopathological findings of the study between groups. [67] Accordingly, no or very little (-), mild (+), moderate (++) and severe (+++) damage were scored (Table 1)

In the OIR group, moderate hemorrhage was observed in the cortex and corpus luteum, while edema findings were observed in the medulla. While leukocyte infiltration from blood vessels into parenchymal tissue was observed at high magnifications, apoptotic and necrotic cells were observed in parenchyma (Fig. 9, Table 1).

Moderate hemorrhage and edema were observed in the OIR+ROL 1 mg/kg group, as in the ischemia-reperfusion group, and apoptotic and necrotic cells were observed in the parenchyma. Mild hemorrhage and edema were observed in the OIR+ROL 3 mg/kg group, and rarely apoptotic and necrotic cells in the parenchyma. While hemorrhage and edema areas were not observed in the OIR+ROL 5 mg/kg group, apoptotic and necrotic cells were not observed in the parenchyma. The general appearance of the OIR+ROL 5 mg/kg group was found to be similar to that of control (Fig. 9, Table 1).

## Discussion

OIR is a complex pathological disease that begins with the cessation of oxygen supply in response to overproduction of free oxygen radicals, then continues with an inflammatory response, and ends with apoptosis and cell death [26]. In this study, The PDE4 inhibitor rolipram was first tested in a rat model of ovarian ischemia reperfusion and its inflammatory levels were determined in each rat group to investigate whether it has a therapeutic effect on OIR. Although rolipram is not currently in active clinical use, its additional anti-inflammatory effect, originally described for its antidepressant properties, is under clinical and experimental investigation in indications for autoimmune disorders [27]. Therefore, the demonstration of the efficacy of PDE4 inhibition in our study suggests that rolipram or similar newly developed PDE4 inhibitors may be used in OIR patients and/or other ischemia reperfusion conditions in the acute intervention phase in addition to other indications. Many studies have shown the beneficial effects of PDE inhibition, in models of I/R injury [28]. In one study, it was shown that PDE inhibitors inhibit the production of proinflammatory genes such as NF-κB and TNF-α in macrophages and microglia by increasing cAMP [29]. Rolipram is known as a selective PDE4 inhibitor and increases intracellular cAMP levels in many tissues and cell types [30]. cAMP is a tightly arranged second messenger involved in different intracellular processes and plays an important role in these processes [31]. Inhibition of PDE4 reduces inflammatory effects by increasing cAMP in these cells [30]. During OIR-induced damage, it causes dysregulation and ovarian damage in the processes of apoptosis and oxidative stress, inflammation by disturbances in the cAMP pathway. Several studies have shown that damage to I/R is reduced by phosphodiesterase inhibitors such as pentoxifylline, aminophylline and rolipram, all of which are known to increase intracellular cAMP levels [32]. In our study, rolipram significantly reduced tissue damage and inflammatory cell count in the OIR group and we also observed an increase in cAMP levels due to increasing doses of rolipram in our results. This can be a result of PDE inhibition by rolipram, for PDE enzymes metabolizes cAMP and when we blocked PDE4 activity cAMP accumulation might be occurred in ROL administered groups. In this context we suggest that there may be a relationship between the cAMP pathway and the anti-inflammatory effects of rolipram.

We also examined the relationship of rolipram with AQP5 to evaluate its protective effects and advantages in OIR. AQPs, are a family of small membrane proteins that transport water across biological membranes. It is important for water transport, many metabolic processes and the survival of living cells [33]. Almost all AQPs except AQP0 and AQP10 were found in the female reproductive system [34]. It has been suggested that AQPs may contrubute to the modulation of ovarian response to exogenous gonadotrophin and positively associated with fertilization rate [35].

AQP1-AQP12 (except AQP0 and AQP10) has been shown to be found in the ovaries and ovaries of rodents [36]. AQP5 in mammals; It was detected in the lung, salivary gland, reproductive system, kidney, eye, gastrointestinal system**.** Also, AQP5 expression has been detected in the oviduct and uterus of many mammalian species, including rats [37–42]. In addition, in a study, it was shown that AQP5 is localized at the protein level on the ovarian epithelial side of the ovarian bursa. Such interesting upregulations of AQP5 in discrete compartments on the ovarian epithelial side of the ovarian bursa indicate their important role in intra-bursa fluid homeostasis [43]. Additionally, a study by Starowicz et al found that while immunofluorescence staining of AQP5 was seen in most oocytes, it was also present in the apical membrane of the epithelial cells of the oviduct ampoule, and after ovulation in rats, AQP5 was shown to play a role in the intracellular movement of water in oocytes and the oviduct ampoule [44]. Previous studies have shown that AQP5 expression was decreased at both mRNA and protein levels in the I/R group compared to the control group [45–47]. Furthermore in an I/R rat study, it was determined that the cAMP-PKA signaling pathway may play a role in the expression of AQP5 protein, and mRNA increases the expression of AQP5 when the cAMP-PKA signaling pathway is activated [48]. Our findings revealed that AQP5 expression decreased in OIR and could prevent the decrease of AQP5 expression after rolipram treatment. Based on these results, we hypothesize that rolipram may affect AQP5 expression by activating the cAMP pathway. However, AQP5 in the ovarian bursa still needs future explanation [43]. In another study, AQP5 immunoreactivity was not detected in mouse ovaries [34]. Therefore, information regarding the role and expression of AQPs in rat female reproductive tissues is still very limited. Therefore, future studies for both rats and humans should be focused on.

During ovarian ischemia, tissues are exposed to destructive pro-inflammatory cytokines and ROS released by inflammatory cells, leading to inflammatory damage [49]. Moreover, unexpectedly, the reperfusion of ischemic tissue following ischemia leads to an elevation in ROS levels due to tissue oxygenation [50]. Therefore; future studies focusing on other possible pathways involving in OIR damage such as oxidative status should be planned. In reperfusion injury, with the influx of oxygen into tissues, ROS and activated neutrophils release pro-inflammatory molecules such as TNF-α and IL-6, which directly cause tissue damage and are potent activators of other neutrophils [51]. Studies have also shown that levels of inflammatory cytokines such as TNF-a and IL-6 are increased in ischemic and/or reperfused tissue [52]. Therefore, I/R is characterized by increased proinflammatory cytokines, including TNF-α and IL-6 [53]. We observed that the IL-6 and TNF-α level in the OIR group was elevated compared to the control. These findings suggest that triggering the inflammatory response may play an important role in the pathogenesis and progression of OIR injury. We found that rolipram reduced protein levels of pro-inflammatory factors, including TNF-α and IL-6, which are used to assess the inflammatory response. Therefore, our findings showed that rolipram could inhibit pro-inflammatory cytokines; This suggested that the anti-inflammatory roles of rolipram in OIR may be related to this mechanism.

Studies have shown that the MAPK signaling pathway and NF-κB signaling pathway, are involved in the regulation of inflammation [54]. Activation of the NF-κB signaling pathway, is functionally associated with its upregulation leading to generation of proinflammatory cytokines. In addition, high levels of other interleukins and TNF-α directly trigger NF-κB signaling, amplification of the initial inflammatory effect [55]. Therefore, many OIR studies have shown that NF-κB levels are increased, suggesting that the NF-κB signaling pathway may increase inflammation and apoptosis and may be the primary oxidative stress-response pathway [56–58]. Similar to our results, we observed that NF-κB levels increased in the I/R group compared to the control. We think that NF-κB and signaling pathways are activated in OIR and rolipram may limit this activation. Therefore, this situation; it suggests that NF-κB may be part of the mechanism by which rolipram exerts its effects.

To appraise the advantages and protective effects of rolipram treatment on OIR, we also examined on the MAPK signaling pathway. Recent studies have clearly demonstrated that MAPKs are associated with increased I/R damage [59]. Studies have shown that the MAPK signaling pathway is involved in a variety of cellular activities, inflammation, innate immunity, proliferation, differentiation, survival and apoptosis [60]. Therefore, stress-activated protein kinases, including c-jun N-terminal kinase (JNK) and p38 MAPK, have been suggested to contribute to the promotion of cell apoptosis and causes overproduction of proinflammatory factors [61]. Recent medical and experimental studies have clearly indicated that MAPKs are linked to an elevated risk of I/R injury [62]. Numerous studies have demonstrated that the protein expression level of MAPK was significantly increased in I/R-associated diseases compared to the control group [63–66]. In our study, we observed that the MAPK level in the OIR group was increased compared to the control and that treatment with increasing doses of rolipram reduces MAPK levels. Based on these results, we think that rolipram may play a protective role in OIR via the MAPK pathway. Thus, regulation of the inflammation-related pathways, the MAPK pathway and the NF-κB pathway may be helpful for OIR.

## Conclusion

The PDE4 inhibitor rolipram can preserve ovary tissue from the harmful effects of OIR damage. The underlying mechanisms may be upregulation of cAMP and downregulation of IL-6/TNF-α/NF-κB/MAPK by rolipram, which may reduce OIR-induced ovarian damage. Rolipram can reduce OIR injury by inhibiting NF-κB, MAPK occurence, reducing TNF-α and IL-6 inflammation levels, and upregulating AQP5 expression. The PDE4 inhibitor rolipram and the different pathways such as IL-6/NF-κB/TNFα/MAPK and cAMP/AQP5 mediating its effects against ovarian torsion damage may be a promising target for ovarian damage or infertility. Therefore, PDE4 inhibition may be a new therapeutic strategy in the treatment of OIR. However, future experimental and clinical studies are needed to detect these results in humans.

## Data Availability

The data that support the findings of this study are available from the corresponding author, EC, upon reasonable request.
